# 4-Hydroxy-3,5-di-tret-butyl cinnamic acid restores the activity of the hippocampal mitochondria in rats under permanent focal cerebral ischemia

**DOI:** 10.22038/IJBMS.2021.58435.12979

**Published:** 2021-11

**Authors:** Dmitry I Pozdnyakov

**Affiliations:** 1 Pyatigorsk Medical and Pharmaceutical Institute (Pyatigorsk, Russia, 357532, av. Kalinina 11)

**Keywords:** Apoptosis, Cerebral ischemia, Cinnamic acid derivatives, Mitochondrial dysfunction, Neuroprotection, Oxidative stress

## Abstract

**Objective(s):**

Ischemic stroke is a disease with complex pathogenesis that requires timely and rational pharmacological intervention. One possible treatment for this condition may be to improve mitochondrial function as part of neuroprotective therapy.

**Materials and Methods:**

Cerebral ischemic damage was reproduced by middle cerebral artery permanent occlusion in Wistar male rats. 4-hydroxy-3,5-di-tretbutyl cinnamic acid was injected intraperitoneally in dose range of 25 mg/kg, 50 mg/kg, and 100 mg/kg. The time of administration was 3 days from the ischemia modeling. Further, changes in the rats’ cognitive functions in the Morris water maze test were evaluated, and the state of mitochondrial function in the hippocampal tissue was studied.

**Results:**

The study showed that the use of the studied compound dose-dependently improved mitochondrial function in the rat hippocampus. At doses of 20 mg/kg and 50 mg/kg, administration of the test substance increased citrate synthase activity by 55.1% (*P*<0.05) and 43.4% (*P*<0.05), respectively and ATP content by 25.7% (*P*<0.05) and 23.9% (*P*<0.05). Also, the intensity of oxidative stress (activity of antioxidant enzymes increase whereas the concentration of TBARS reduces) and apoptosis (calcium content, concentration of apoptosis-inducing factor, and caspase-3 activity decrease; latent time of mitochondrial transition permeability pore opening increase) decreased against the background of administration of the test compound. At a dose of 100 mg/kg, the studied compound showed less effectiveness.

**Conclusion:**

Administration of 25 mg/kg and 50 mg/kg 4-hydroxy-3,5-di-tretbutyl cinnamic acid demonstrated neuroprotection action on hippocampal cells under the conditions of irreversible brain ischemia.

## Introduction

Ischemic stroke is one of the main reasons for mortality and primary disability in the adult population (1). Global statistics show that despite the progress achieved in the prevention, diagnosis, and treatment of ischemic stroke, this pathological condition is still a rather serious problem in modern medicine. More than 80 million cases of ischemic stroke are reported annually, and more than 25 million are fatal in the acute phase of the disease (2). The percentage of primary disability due to stroke also remains high; more than 40% of stroke survivors are unable to return to work and lose social adaptation skills, which inevitably leads to a decreasing in the disability-adjusted life years (DALYs). According to a study by Johnson *et al*. published in Lancet Neurology, 116.4 million DALYs were lost due to ischemic stroke in 2016 alone (3). In many ways, the high level of disability in stroke patients is mediated by damage to the hippocampus and development of cognitive impairment, combined with post-stroke dementia (PSD). Renjen* et al. *showed that ischemic stroke increases the risk of developing dementia by 4–12 times, and PSD *per se* is observed in a fairly wide range—from 6% to 55% of cases and, which is very important, PSD increases the probability of re-stroke almost twice as much (4). In this regard, numerous attempts have been made to reduce the risk of developing PSD by developing and implementing appropriate pharmaco-therapeutic strategies in clinical practice. It was found that the use of recombinant plasminogen activator (rt-PA) preparations in patients with mild ischemic stroke reduced the probability of developing cognitive impairment (5). However, the administration of rt-PA drugs, the “gold standard” of the ischemic stroke treatment, has a significant number of limitations to its use with strict patient selection, a small “therapeutic window” and the risk of developing hemorrhagic complications (6). Alternative approaches to PSD correction were also developed. Zhang* et al. *(2019) demonstrated that an appropriate rehabilitation program, without active pharmacological intervention, could effectively reduce post-stroke cognitive impairment (7). At the same time, neuroprotection may be one of the promising areas of PSD treatment (8). Recently, there has been a growing scientific interest in the targeted search of neuroprotective agents as adjuvant therapy of ischemic stroke, which can reduce the risks associated with the use of tr-PA drugs, as well as increasing the effectiveness of thrombolytic therapy (9).

Among potential neuroprotective agents, anti-oxidant agents stand out, which, through a number of mechanisms, can significantly reduce the degree of brain neuron damage during ischemia (10). The multifunctional mechanism of action of anti-oxidants includes the effect on changes in mitochondrial function, which in turn can contribute to both the enhancement of the neuroprotective effect of anti-oxidants and an independent aspect of neuroprotection, which was shown by the example of Coenzyme Q_10 _(CoQ_10_) (11). 4-hydroxy-3,5-di-tret-butyl cinnamic acid (ATACL code) is widely represented in the pulp of pumpkin fruits, and can also be obtained synthetically. Previous studies showed that the use of the ATACL compound under cerebral ischemia contributed to a decrease of the brain necrosis area, restoration of cerebral hemodynamics, and a decrease in blood thrombogenicity (12). However, despite the potentially high neuroprotective potential of this compound, the mechanism by which the neuroprotective effect of 4-hydroxy-3,5-di-tret-butyl cinnamic acid is realized has not been established. Also, the effect of this compound on post-stroke CNS changes, including the development of cognitive impairments, was not evaluated, which was the aim of this study: to evaluate the effect of 4-hydroxy-3,5-di-tret-butyl cinnamic acid on the activity of mitochondria in the hippocampus of rats under conditions of permanent cerebral ischemia.

## Materials and methods

***Laboratory animals***

In this work, 120 male Wistar rats (200–220 grams, 3 months old) were used. The rats were obtained from Rappolovo experimental animal hatchery (Russia, Leningrad region) and during the experiment were kept under controlled conditions in the laboratory of living systems of the Pyatigorsk Medical and Pharmaceutical Institute. Conditions of detention: 22 ± 2 °C temperature, 60 ± 5% humidity , with a 12-hour change in the daily cycle. The rats were kept 5 animals per macrolon cage on a granular hardwood bedding with free access to water and food. The concept of study and animal manipulations were regulated by Directive 2010/63 / EU of the European Parliament and of the council on the protection of animals used for scientific purposes, September 22, 2010, and ARRIVE guidelines (13). The concept of research was endorsed by the ethics committee of PMPI (protocol no. 20, dated 16 May, 2020).

***Chemicals***

The test compound, 4-hydroxy-3,5-di-tret-butyl cinnamic acid (under cipher ATACL), was obtained at the Department of Organic Chemistry of the Pyatigorsk Medical and Pharmaceutical Institute. The structure was confirmed by NMR spectroscopy. As the reference drug, ethylmethylhydroxypyridine succinate (Mexidol®, Pharmasoft, Russia) was used. The reagents used in this work (unless otherwise indicated) were provided by Sigma-Aldrich (Darmstadt, Germany).

***Study design***

**Figure 1:**
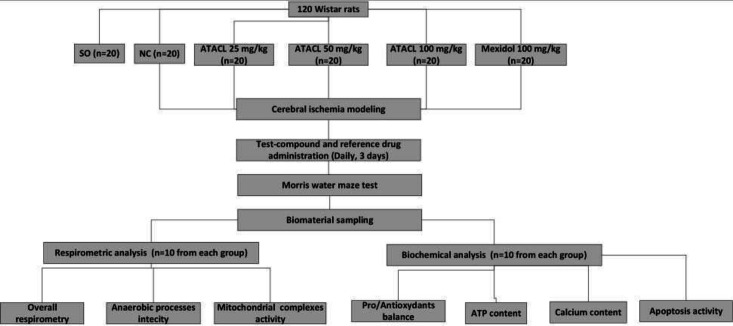
Design of the research

When setting up the experiment, the following groups were formed: SO, sham-operated rats (consecutive surgical procedures without arterial coagulation were applied to this group); NC, negative control group (not receiving pharmacological support); a group of rats that received the test compound in the form of an aqueous fine-dispersed suspension, manufactured without the use of adjuvants at doses of 25 mg/kg, 50 mg/kg, and 100 mg/kg; a group of animals that received 100 mg/kg of a reference drug (12). Twenty individuals formed one experimental group. The study design provided for the therapeutic administration of the analyzed compound and Mexidol. The administration was carried out immediately after awakening the animals and once a day for three days, intraperitoneally (the test compound was dissolved in water for injection). On the 4^th^ day, the spatial memory of rats was assessed in the Morris water maze test. After that, animals were decapitated under anesthesia. Further, the hippocampus was isolated, which was used as an analyzed biomaterial. In ten rats from the group, the parameters characterizing the change of the cellular respiration processes were determined, and in the 10 remaining animals, the change in the pro/anti-oxidant balance and the activity of apoptosis were assessed. The design of the study is represented in Figure 1.

***Experimental model of cerebral ischemia***

Brain ischemia in rats was reproduced by the method of permanent middle cerebral artery occlusion. In anesthetized rats (chloral hydrate (Acros Organics) 350 mg/kg, intraperitoneally), the surface lower right eye was depilated, the cutaneous tissues were dissected and the muscles were separated. The zygomatic process was removed, exposing the skull. Above the intersection of the middle cerebral artery and the olfactory tract, a trepanation hole was made and the artery was coagulated. The wound was sutured in layers with a polydioxanone-based thread (USP1). The wound was cleared by an antiseptic solution - benzyldimethyl (3-myristoilamine) propyl) ammonium chloride monohydrate); 0.01% solution, INFAMED corp.). Animals were placed under a heating lamp until awakening (14).

***Morris water maze test***

The Morris water maze device is a water arena (diameter of 1.5 m) with a wall height of 0.6 m and a movable platform with a diameter of 0.1 m. During the study, the arena was filled with water to the 50 cm level, after which the water was tinted by a blue dye. Before cerebral ischemia modeling, rats were trained in the testing procedure: within 2 min the animals were allowed to find a platform, provided no execution of the task, rats were moved to the platform for 10 sec, after that, the testing was repeated. The training lasted for 5 days. After reproducing the ischemia, the test was repeated. The platform was also removed from the device. The latency period of reaching the platform in seconds, the distance that the animal spent on finding the platform in meters, and the average speed were recorded. The experiment was recorded and processed using the Minotaur software (Neurobiotics, Russia) with infrared monitoring of activity (15).

***Biomaterial sampling, mitochondrial isolation***

In this work, as a biomaterial, the hippocampus of rats was used. All isolation procedures were performed at 4 °C. In ten animals out of each group, the hippocampus was homogenized in a buffer solution containing sodium chloride, sodium hydrophosphate, potassium chloride, and potassium dihydro phosphate (in 1:7 ratio with final pH of 7.4) in a homogenizer of a mechanical type and centrifuged under 10000g mode for 5 min. The received supernatant was used in an ELISA study and for anti-oxidant activity determination. In the other 10 rats, the hippocampus was homogenized in a solution of following composition: EGTA (1 mmol/l) + mannitol (215 mmol/l) + sucrose (75 mmol/l) + bovine serum albumin solution (0.1 g, in 100 ml of water) + HEPES buffer solution (20 mmol/l, with pH of 7.2). For mitochondrial isolation, the received homogenate was centrifuged under 1100 g for 2 min. The resulting fraction in the amount of 700 µl was transferred into Eppendorf tubes and mixed with 75 µl of 10% percoll solution and centrifuged at 18000 g for 10 min. The precipitate was resuspended in 1 ml of the solution for isolation, and centrifugation was repeated for 5 min at 10,000 g. The resulting fraction was removed for respirometric analysis and measurement of mitochondrial permeability transition pore opening (16).

***Cell respirometry***

Cell respirometry was carried out on an AKPM 1-01L laboratory respirometer (Alfa Bassens, Russia) with determination of the change of O_2_ consumption (OCR) against the background of the injection of mitochondrial respiration uncouplers into the analyzed medium. As uncouplers in this work, sodium azide (20 mmol/ml); oligomycin (1 µg/ml); rotenone (1 µmol/ml); and FCCP (1 µmol/ml) were used. Based on the obtained values of the change in oxygen consumption, the following parameters were calculated: ATP-generating capacity, maximum respiration rate, and respiratory capacity. The activity of glycolysis processes was assessed by the change of OCR in the medium against the background of the addition of an oxidation substrate - glucose (15 mmol/ml), as well as oligomycin and sodium azide. The activity of anaerobic processes was measured by changes of the following parameters: the glycolytic capacity, glycolysis intensity, and glycolytic reserve. The mitochondrial respiratory chain component activity (complexes I, II, IV, and V) was studied by adding the corresponding oxidation substrates to the analyzed medium: pyruvate (10 mmol/ml), 1 mmol/ml of malic acid, succinic acid (10 mmol/ml), 2 mmol/ml of ascorbate, 1 mmol/ml of ADP, and 0.5 mmol/ml of TMPD as described by Connolly* et al., *2018 (17). The complex I activity was investigated by the changes of OCR when the pyruvate+malate mixture was used as oxidation substrate. The complex II activity was tested by the changes of OCR when succinate and oligomycin were added to the analyzed solution. The complex IV activity was tested by OCR difference after adding the mixture of substrates TMPD+rotenone+ascorbate. The complex V activity was tested by OCR changes after rotenone and ADP were added to the analyze solution. The complex III activity was assessed using the spectrophotometric method by increasing the absorbance of the incubation medium with 1 M succinate solution + 0.5 M cytochrome C solution + 0.2 M KCN solution + 10 mmol rotenone solution and 10 μl of the analyzed sample at 550 nm (18). OCR was determined in ppm and expressed in terms of protein concentration in the analyzed sample. Protein content was evaluated by the Bradford method (19).

***Measurement of mitochondrial transition permeability pore latent time of opening (mPTP)***

In this study, a spectrometric approach was used. The analyzed medium contained 0.05 ml of tested supernatant, 1 μmoles/ml cyclosporin A solution (0.05 ml), and 200 mmoles of KCl solution (20 μl). The resulting mixture was adjusted to 200 μl by a HEPES buffer solution pH of 7.4. Then the absorbance was recorded at a wavelength of 540 nm at room temperature for 25 min. The latent time of mPTP opening was determined in seconds required to decrease the absorbance of the medium by 0.2 units (20)

***Measurement of citrate synthase activity***

The enzyme activity was evaluated by a spectrophotometric approach which is based on the measurement of absorbance of 5.5’-di-thiobis- 2-nitrobenzoic acid degradation products in a medium containing oxaloacetate and acetyl-CoA. The reaction mixture contained: 100 mM DTNB solution, 100 mM acetyl CoA solution, Triton-X - 0.1% solution, 4 μl of the analyzed supernatant ,and Tris-HCl buffer solution with pH 7.8 to 100 μl. The absorbance of the mixture was tested at the wavelength of 412 nm for 3 min. The citrate synthase activity was determined by the change in the absorbance of the medium and was expressed in U/mg of protein. The Bradford method was used to determine protein concentration (21).

***Measurement of the Ca*** ^2+^ ***concentration***

The Ca^2^^+^ content in the tested samples was investigated by the Fura-2/AM fluorescence. The incubation medium contained 100 μl of the analyzed sample and Fura-2/AM in equal content. The intensity of the fluorescent signal was determined at excitation/emission wavelengths of 340 nm/380 nm and a 510 nm filter. Fluorescence was recorded on a Hitachi MPF-4 spectrofluorometer. The calcium content in the samples was determined in terms of protein concentration. Calcium concentration was estimated in fresh samples of biomaterial without freezing (22).

***Measurement of the*** ***Н***_2_ ***О***_2_ ***concentration***

The concentration of Н_2_О_2_ was determined by fluorescence analysis using a standard Amplex Red kit (Thermo Fisher Scientific). The Amplex Red reagent forms fluorescent resorufin in reaction with hydrogen peroxide (excitation/emission = 570/585 nm). The analysis followed the manufacturer’s instructions. The content of hydrogen peroxide was estimated in nmol/ml.

***Measurement of 2-thiobarbituric acid reactive substances (TBARS)***

The TBARS concentration was determined spectrophotometrically by detection of condensation products of aryl aldehydes with 2-thiobarbituric acid at 532 nm. 100 μl of the analyzed biomaterial was mixed with an equivalent amount of 1.7% trichloroacetic acid solution and incubated for 5 min. The received mixture was centrifuged for 10 min at 1000g. The supernatant was transferred into Eppendorf tubes and a 0.88% TBA solution was added and incubated in a boiling water bath for 15 min. The absorbance of the obtained colored solutions was recorded at 532 nm. The concentration of TBARS was calculated by the value of the molar extinction coefficient of malondialdehyde (1.56 × 10^5^ l × mol^-1 ^× cm^-1^), the results were expressed in nmol/mg protein (23).

***Investigation of catalase activity***

Catalase activity was determined using the spectrophotometry method by the rate of hydrogen peroxide destruction. The amount of hydrogen peroxide was determined in reaction with an ammonium molybdate aqueous solution 4% concentration. The color intensity of the reaction product was evaluated at 410 nm. Catalase activity was determined by the difference between the absorbance of the experimental and blank samples, using the molar extinction coefficient of hydrogen peroxide equal to 22.2×10^3^ mm^-1^ cm^-1^ and expressed in U/mg of protein (24).

***Measurement of superoxide dismutase activity***

The activity of superoxide dismutase (SOD) was evaluated by the xanthine-xanthine oxidase method based on the reaction of the superoxide radical dismutation that formed during the oxidation of xanthine and reduction of 2- (4-iodophenyl) -3- (4-nitrophenol) -5-phenyltetrazolium chloride (NTC). The incubation medium contained: xanthine (0.05 mmol/l), NTC (0.025 mmol/l), xanthine oxidase (80 U/L), EDTA (0.94 mmol/l), and CAPS (40 mmol/l). The absorbance of the mixture was recorded at 505 nm. SOD activity was recalculated on protein concentration in mg (25).

***Investigation of glutathione peroxidase activity***

Glutathione peroxidase (GPx) activity was determined by the spectrophotometry method in the conjugated glutathione reductase reaction according to the decreased NADPH concentration. The incubation medium contained: 1 mmol/l EDTA, 50 mM PBS (pH 7.4), 1 U/ml glutathione reductase, 20 mmol/l NADPH, and 1 mmol/l GSH. The absorbance was recorded at 340 nm. The reaction was started by adding a substrate (cumene hydroperoxide, 1.5 mmol/l) and was carried out at a temperature of 25 °C. GPx activity was expressed in U/protein mg (26).

***ELISA study***

In this study, the change in the content of adenosine triphosphate (ATP), apoptosis-inducing factor (AIF), and caspase-3 was assessed by the ELISA method in the supernatant of the animal’s hippocampus. Assay kits were obtained from Cloud Clone Corp. (Houston, USA). The determination process, sample preparation, and processing of the obtained spectrophotometric signal corresponded to the manufacturer’s recommendations.

***Statistical analysis***

The analysis of the obtained data was carried out using the Statistica 6.0 software. Results were expressed as M ± SEM (mean ± standard error of the mean). The statistical analysis was performed by the ANOVA method with the Newman-Keuls *post hoc* test in the case of data obeying the law of normal distribution and the Kruskal-Wallis test when the data was distributed other than normally. The normality of the distribution was checked using the Shapiro-Wilk test. Differences were considered significant at the *P*<0.05 significance level.

## Results

***Influence of test compound on behavior change of rats in the Morris water maze test***

**Figure 2:**
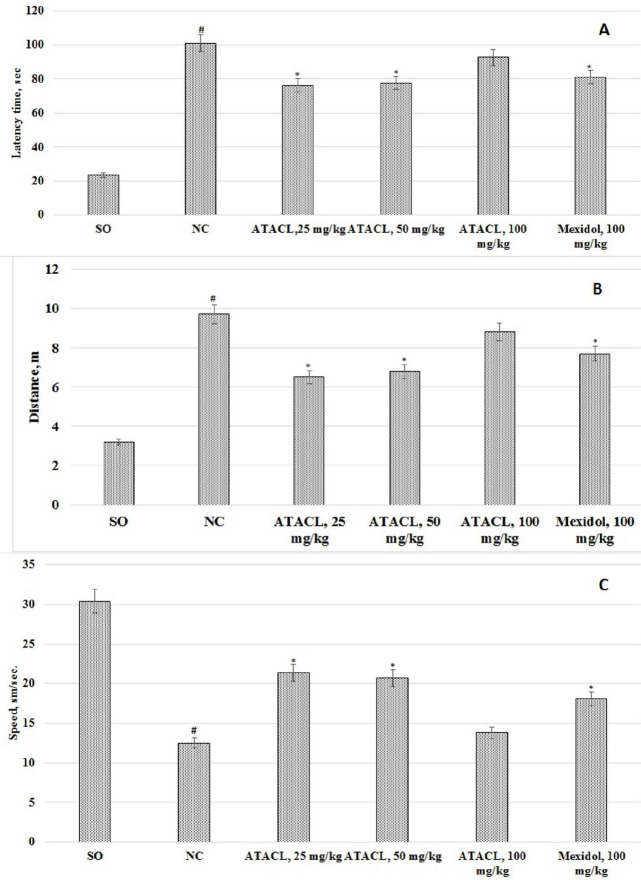
Effect of 4-hydroxy-3,5-di-tret-butyl cinnamic acid and referent on behavior change of rats in the Morris water maze test

Note: A – latent period of the platform; B – distance; C – average speed; # - reliably relative to the SO rats (*P*<0.05, ANOVA by post-processing with Newman-Keuls test); * - # - reliably relative to the NC rats (*P*<0.05, ANOVA by post-processing with Newman-Keuls test)

The results are represented in Figure 2. In the experimental NC group of rats the latent time of task performance was higher by 4.3 times, swimming distance by 3.0 times, with a decrease in the average speed of movement by 58.9% relative to SO rats. The ATACL compound administration promoted a reduction of the task implementation latent time and the covered distance at a dose of 25 mg/kg by 24.5% and 33.0%, respectively, at a dose of 50 mg/kg by 23.1% and 30.1%, respectively (data in relation to the NC group). Also, in the rat group that was treated by ATACL at doses of 25 mg/kg and 50 mg/kg, there was an increase in average swimming speed by 71.2% and 65.6%, respectively. Administration of the reference medicine to the animals promoted a reduction of the task performance latent time and the covered distance, with an increase in the average speed by 19.5%, 20.6%, and 44.8%, respectively. Thus administration of the compound under the ATACL cipher in a dose of 100 mg/kg does not change the behavior of animals in the Morris water maze test. Besides, in rats that were treated by ATACL (25 and 50 mg/kg), the latent time for finding the platform and the covered distance were lower, and the average swimming speed was correspondingly higher than in animals that received the reference drug.

***Influence of test compound on cellular respiration in the supernatant of the animals’ hippocampus***

**Figure 3:**
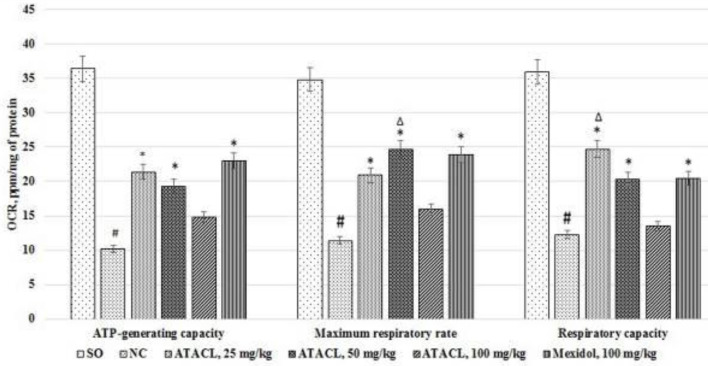
Influence of 4-hydroxy-3,5-di-tret-butyl cinnamic acid and referent change processes cellular respiration in the supernatant of the hippocampus in rats

Note: # - reliably relative to the SO rats (*P*<0.05, v); * - # - reliably relative to the NC rats (*P*<0.05, ANOVA by post-processing with Newman-Keuls test); Δ - reliably relative to the group of rats treated by Mexidol (*P*<0.05, ANOVA by post-processing with Newman-Keuls test)

The results of this research block are shown in Figure 3. Analysis allowed that in the NC group of animals ATP-generating capacity, maximal respiration level, and respiratory capacity decrease by 72%, 67.2%, and 65.7%, respectively (data are shown relative to SO rats). Administration of the Mexidol, elevate the ATP-generating capacity by 125.5%, the maximal respiration level by 109.6%, and the respiratory capacity by 66.7% in relation to the rats of the NC group. The use of the 25 mg/kg and 50 mg/kg of ATACL compound contributed to a significant increase in the parameters of cellular respiration: the ATP-generating capacity increased by 109.8% and 89.2%, the maximal respiration level by 83.3% and 116.7%, and respiratory capacity by 100.8% and 65.0%, respectively. At the same time, the maximum level of respiration in rats treated with 50 mg/kg of ATACL compound and the respiratory capacity in animals that received the test compound at 25 mg/kg dose were higher than analogous parameters in the group of rats receiving the reference. It is significant that at a dose of 100 mg/kg with intraperitoneal administration, the ATACL compound does not change the processes of cellular respiration.

***Influence of test compound on changes in the activity of anaerobic processes in the supernatant of the animals’ hippocampus***

**Figure 4:**
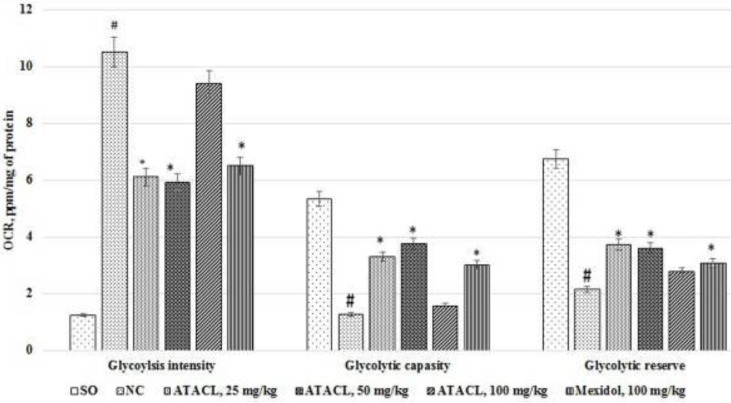
Effect of 4-hydroxy-3,5-di-tret-butyl cinnamic acid and the referent on changes in the activity of anaerobic processes in the hippocampal supernatant in rats

Note: # - reliably relative to the SO rats (*P*<0.05, ANOVA by post-processing with Newman-Keuls test); * - # - reliably relative to the NC rats (*P*<0.05, ANOVA by post-processing with Newman-Keuls test)

The results of assessing the anaerobic process activity in the hippocampus are shown in Figure 4. For example, in animals that did not undergo pharmacological intervention, the intensity of glycolysis was 8.4 times higher than that in SO rats, with a reduction of glycolytic capacity and reserve by 76.4% and 68.2%. With the administration of the referent, a decrease of the glycolysis intensity in comparison with the NC group of rats by 38.1% was noted, besides the glycolytic capacity increase by 138.9% and the glycolytic reserve by 43.6%. The use of 25 mg/kg ATACL compound led to reduction of the glycolysis intensity with elevation of glycolytic capacity and glycolytic reserve by 41.8%, 161.1%, and 74.3%, respectively. In the group of rats that were treated by 50 mg/kg of the test compound, a reduction of the glycolysis intensity by 43.6%, and elevation of the capacity of glycolysis and glycolytic reserve by 198.4% and 68.2%, respectively was noted (indicators are given in relation to the NC group of animals). Besides that, statistically significant differences between the NC group of rats and animals receiving 100 mg/kg ATACL were not found.

***Influence of test compound on changes of the mitochondrial respiratory chain complex activity in the supernatant of the animals’ hippocampus***

**Figure 5:**
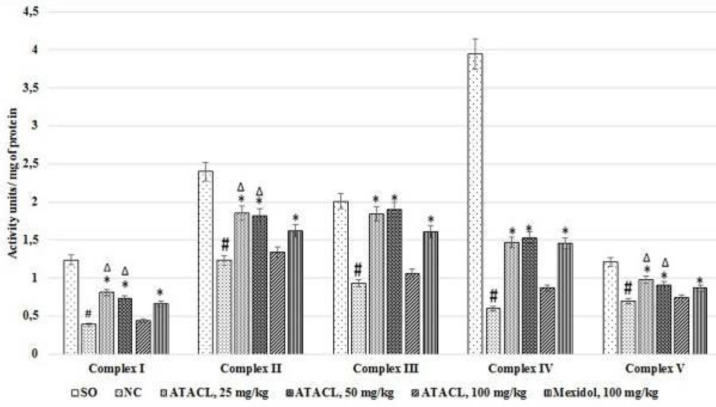
Effect of 4-hydroxy-3,5-di-tret-butyl cinnamic acid and referent on changes of the activity of mitochondrial respiratory chain complexes in the hippocampal supernatant of rats

Note: # - reliably relative to the SO rats (*P*<0.05, ANOVA by post-processing with Newman-Keuls test); * - # - reliably relative to the NC rats (*P*<0.05, ANOVA by post-processing with Newman-Keuls test)

The results of this experimental block are shown in Figure 5. In the NC group of animals in relation to the SO group, a decrease of the mitochondrial complexes I, II, III, IV, and V activity by 68.6%, 48.8%, 53.7%, 84.8%, and 42.2%, respectively was observed. Thus, administration of Mexidol contributed to elevation of the respiratory complex activity relative to the NC animals by 69.2%, complex I; 31.7%, complex II; 73.1%, complex III; 143.3%, complex IV; and 24.3%, complex V. In animals that were treated by 25 mg/kg and 50 mg/kg of the test compound, an increase of the mitochondrial complex activity was also observed (in comparison with the NC group), while complex I activity increased by 107.7% and 87.2%; complex II by 50.4% and 48.0%; complex III by 97.9% and 104.3%; complex IV by 145.0% and 155.0%; complex V by 40.0% and 30.0%, respectively. It should be noted that activity of complexes I, II, and V in the group treated by 25 mg/kg and 50 mg/kg of ATACL compound were higher than those in rats that were treated by the reference. Administration of the 100 mg/kg of test compound did not change the mitochondrial complex’s activity (no statistically significant differences in relation to the NC rats were found).

***Influence of ATACL compound on changes in citrate synthase activity and ATP concentration in the supernatant of the animals’ hippocampus***

**Figure 6:**
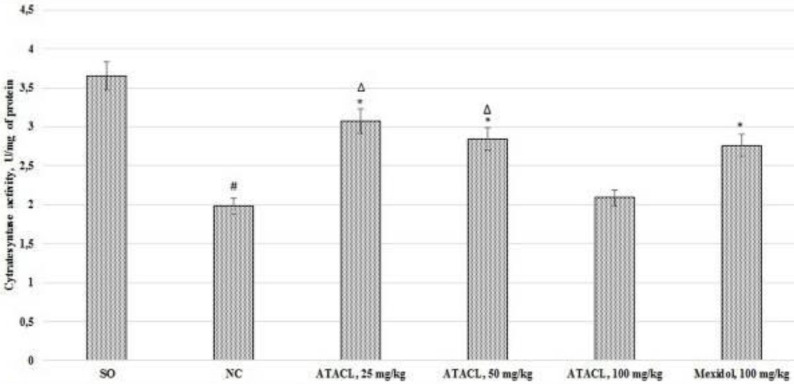
Effect of 4-hydroxy-3,5-di-tret-butyl cinnamic acid and referent on changes in citrate synthase activity in the hippocampal supernatant of rats

Note: # - reliably relative to the SO rats (*P*<0.05, ANOVA by post-processing with Newman-Keuls test); * - # - reliably relative to the NC rats (*P*<0.05, ANOVA by post-processing with Newman-Keuls test); Δ - reliably relative to the group of rats treated by Mexidol (*P*<0.05, ANOVA by post-processing with Newman-Keuls test)

**Figure 7:**
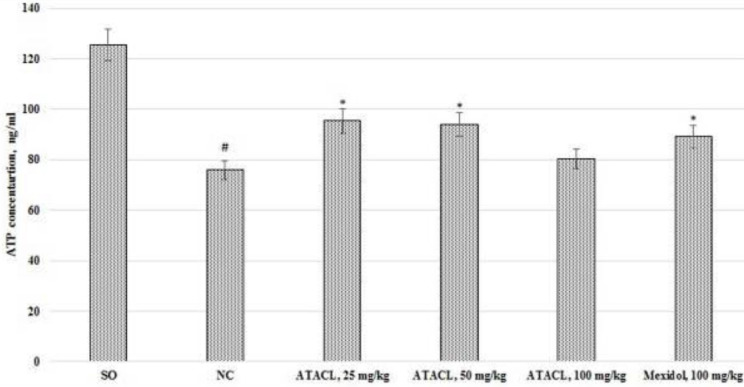
Effect of 4-hydroxy-3,5-di-tret-butyl cinnamic acid and the referent on changes of the concentration of ATP in the hippocampal supernatant in rats

The results of the citrate synthase activity evaluation are shown in Figure 6, the concentration of ATP is shown in Figure 7. In the course of evaluation of the change of the activity of the citrate synthase, it was found that the catalytic properties of this enzyme in the NC group decreased by 45.8% in relation to the same indicator of SO rats, also NC group of rats showed decreasing of ATP concentration by 39.6%. The use of a reference drug contributed to the increase of citrate synthase activity and ATP content by 39.4% and 17.9%, respectively. Besides, by administration of the test compound to animals at doses of 25 mg/kg and 50 mg/kg, an increase of the activity of the citrate synthase by 55.1% and 43.4% was observed, as well as increased level of ATP by 25.7% and 23.9%, respectively. The citrate synthase activity was higher in the animal group that received the test compound at doses of 25 and 50 mg/kg than in rats that were treated by the reference. Thus there were no statistically significant changes of citrate synthase activity (in relation to the NC rats) in rats that were treated with 100 mg/kg of ATACL.

***Influence of test compound on changes in the pro/anti-oxidant balance in the supernatant of the animals’ hippocampus***

The results of evaluating the effect of ATACL and the reference drug on the change in the pro/anti-oxidant state are presented in Table 1. In the course of this section of experimental work, in the NC group of rats, a decrease (relative to the SO animals) in the activity of the endogenous anti-oxidant enzymes SOD by 57, 6%, catalase by 52.2%, and GPx by 38.7%, with an increase in the concentration of H_2_O_2_ and TBARS by 2.4 and 3.6 times, respectively were noted. The use of the referent increased the activity of SOD, catalase, and GPx in relation to the NC group of rats by 22.4%, 14.3%, and 40.2%, while the content of H_2_O_2_ and TBARS decreased by 32.7% and 39.0%, respectively. Besides, administration of the test compound at a dose of 25 mg/kg promoted an increase of SOD activity by 38.6%, catalase by 47.0%, and GPx by 24.9%, as well as a decrease of the prooxidant concentration; H_2_O_2_ by 36.0% and TBARS by 32.2%. Similarly, the use of ATACL compound at a dose of 50 mg/kg led to the restoration of pro/anti-oxidant balance, which was expressed in increase of the SOD, catalase, and GPx activity by 46.1%, 47.0%, and 27.8%, respectively, with a decrease of H_2_O_2_ and TBARS content by 35.4% and 17.5%. It should be noted that activity of SOD and GPx in rats treated by the ATACL compound was higher than in animals treated by the reference. In addition, 100 mg/kg of ATACL compound did not change the activity of endogenous anti-oxidant defense enzymes, but statistically significantly increased the content of H_2_O_2_ by 31.0% and TBARS by 41.0%, relative to the NC rats.

***Effect of the test compound on the change of apoptosis activity in the supernatant of the animals’ hippocampus***

**Figure 8:**
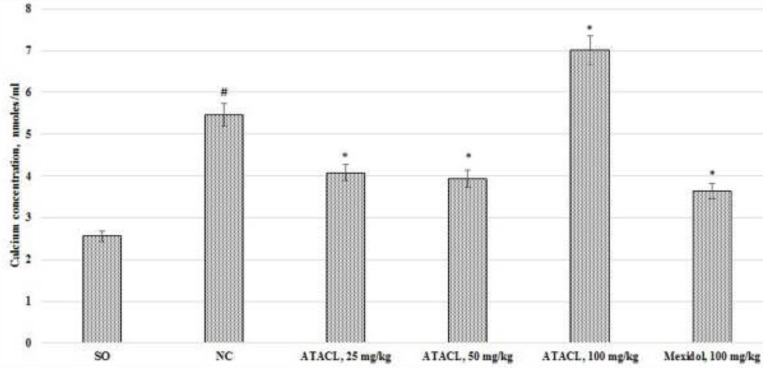
Effect of 4-hydroxy-3,5-di-tret-butyl cinnamic acid and referent on changes in calcium concentration in the hippocampal supernatant in rats

**Figure 9:**
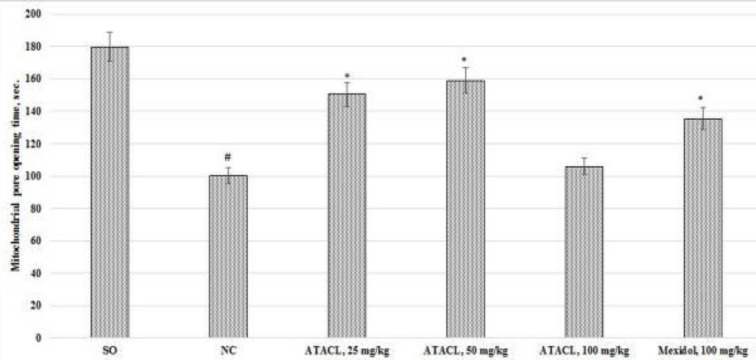
Effect of 4-hydroxy-3,5-di-tret-butyl cinnamic acid and the referent on the change of the latent opening time of mPTP in the hippocampal supernatant of rats

**Figure 10:**
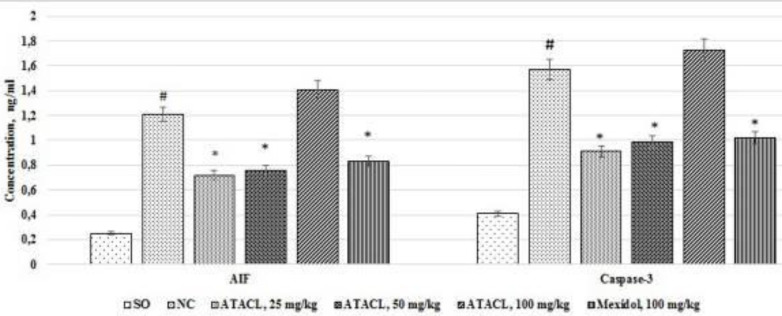
Effect of 4-hydroxy-3,5-di-tret-butyl cinnamic acid and referent on changes of the AIF and caspase-3 concentration in the hippocampal supernatant of rats

The activity of apoptosis reactions in the supernatant of the hippocampus in rats was evaluated by changes in the ionized calcium content (Figure 8), the mPTP latent time of opening (Figure 9), and the content of AIF and caspase-3 (Figure 10). As a result, it was registered that in the NC group of animals, in comparison with SO rats, eluviation of the ionized calcium content, 2.1 times; reduction of the mPTP opening latent time, by 44.2%, as well as increase in the content of AIF and caspase-3 by 4.8 and 3.8 times, respectively was noted. In the group that was treated by Mexidol a decrease (relative to the negative control group) in the concentration of calcium, AIF, and caspase-3 by 36.6%, 31.4%, and 35.0%, respectively, with increase of the latent time of mPTP opening by 35.0% was noted. Use of the ATACL compound at a dose of 25 mg/kg also led to a decrease in the intensity of apoptotic reactions, which was expressed in decrease of calcium content by 25.4%, AIF by 40.5%, caspase-3 by 42.0%, and an increase in latency time of mPTP opening by 50.0%. At the same time, in animals receiving the ATACL compound at a dose of 50 mg/kg, relative to the NC group of rats, a decrease in the concentration of AIF and caspase-3 by 37.2% and 36.9%, respectively, ionized calcium by 28.2%, with an increase in latent time of opening of mPTP by 58.4% was noted. It should be noted that the use of 100 mg/kg of the ATACL compound promoted an increase in the calcium content in the supernatant of the hippocampus of animals by 29.8%, as well as an insignificant increase in the concentration of AIF and caspase-3 in relation to the same indexes of the NC group.

## Discussion

Neuroprotection involves protection of neurons from the action of a damaging factor through the elimination of certain pathogenic mechanisms of neuronal damage and represents a promising direction in the adjuvant therapy of ischemic stroke (27). It should be noted that, despite the lack of translational success, the scientific and practical interest in neuroprotective agents remains at a sufficiently high level, which determines the relevance of the search for new strategies for neuroprotection, as well as the neuroprotectors themselves. (28). New areas of neuronal protection include suppression of excitotoxicity, oxidative stress, and increasing the intensity of “repair of neurons”, which can be achieved by restoring metabolic reactions in the ischemic penumbra zone (29). It is known that a decisive role in the cell metabolism, including neurons, is played by a change in mitochondrial function, which is reflected at once in several leading cellular processes: ATP synthesis in oxidative phosphorylation reactions, redox state, and regulation of apoptotic signal (30). To date, it has been established that mitochondrial dysfunction is an inherent component of pathogenesis of the ischemic damage of the brain, while mitochondrial alteration can negatively affect several cell functions at once, namely, a decrease in the synthesis of high-energy phosphates, an apoptotic cascade is activated and lipid peroxidation processes are enhanced (31). As a rule, mitochondrial damage is observed when the level of cerebral blood flow reaches less than 23 ml/100 g tissue/min and is mediated by a decrease in oxidative metabolism due to a lack of oxygen. At the same time, the electronic flux at the level of complexes I and III is directed not along the main, but along the secondary path, which leads to an increase in ROS production (32). At the same time, a decrease in the intensity of oxidative phosphorylation reactions and, accordingly, ATP synthesis, leads to the activation of the pro-apoptotic signal, an increase in the intracellular calcium content, and the opening of mPTP, promoting AIF release and activation of caspase-associated apoptosis (33). In this regard, this study was carried out on a promising neuroprotective molecule, 4-hydroxy-3,5-di-tret-butyl cinnamic acid, on mitochondrial function modifications in rats under cerebral ischemia conditions. In previous studies, it was found that the use of this compound helps to reduce the zone of ischemic necrosis of the brain and restore the level of cerebral blood flow in experimental permanent ischemia. However, the mechanism by which the test compound reduces the extent of neuronal damage has not been established. Also, given that the most formidable complication of ischemic stroke is acute PSD, which is associated with damage to the hippocampus, this study focused on assessing the effect of ATACL on changes in mitochondrial function in the hippocampus of animals under permanent brain ischemia. In this study, preference was given to the model of permanent middle cerebral artery occlusion because, as McBride & Zhang (2017) point out, this experimental pathology most fully reflects the pathogenetic mechanisms of brain damage observed in the clinic in humans (34). The “STAIR” working group (Recommendations for standards regarding preclinical neuroprotective and restorative drug development) also recommends a model of permanent middle cerebral artery occlusion for the study of neuroprotective agents. In addition, one of the STAIR recommendations that allows translational success is to compare potential neuroprotective agents with drugs already introduced into medical practice. In this regard, the reference drug in this work was ethylmethylhydroxypyridine succinate (Mexidol®), which demonstrates a high level of efficacy, both in experimental pharmacology (35) and in clinical conditions, when used as a neuroprotective adjuvant to the base therapy of ischemic stroke (36). As a result of the study, it was found that the use of ATACL in doses of 25 mg/kg and 50 mg/kg with parenteral administration contributed to the restoration of energy-producing mitochondrial function in the hippocampus of ischemic animals, which was reflected in the normalization of the processes of aerobic/anaerobic metabolism and the maintenance of the mitochondrial respiratory chain complexe activity. Besides, with the administration of 4-hydroxy-3,5-di-tret-butyl cinnamic acid in the indicated doses, a decrease in the intensity of apoptosis reactions was noted, as evidenced by a decrease in the content of pro-apoptotic molecules AIF and caspase-3. At the same time, the antiapoptotic effect of the test compound may be associated with a decrease in the concentration of intracellular calcium and an increase in the latent time of mPTP opening, as main predictors of an apoptotic event (37). It is known that mPTP is a channel of the inner mitochondrial membrane, which is also formed by the c-ring residues F_1_F_0_ of ATP-synthase, leading to the release of apoptotic proteins and induction of apoptosis (38). In many ways, formation of mPTP is a consequence of the dissipation of mitochondrial bioenergetic processes and an increase in the concentration of calcium, which is supported by ROS. As a rule, the formation of mPTP is a no-return point, after which the process of cell death is irreversible (39). The decrease in the latent time of mPTP opening observed against the background of the administration of 4-hydroxy-3,5-di-tret-butyl cinnamic acid may be associated with a decrease in the concentration of ionized calcium, as well as with the stabilization of F_1_F_0_ ATP-synthase, which prevents its dimerization into one of the mPTP components. As a result, mitochondria remain viable, ATP synthesis is stabilized, and concentration of AIF and caspase-3 decreases, preventing cell death through apoptosis.

In addition, when the test compound was used in doses of 25 and 50 mg/kg (intraperitoneally), it reduced the intensity of oxidative stress reactions. So, against the background of administration of ATACL in the indicated doses, a decreased intensity of lipid oxidation processes and ROS generation was noted, as evidenced by a reduction in the concentration of TBARS and H_2_O_2_. Also, the use of the test compound led to an evaluation of the activity of the main anti-oxidant enzymes SOD, catalase, and GPx. At the same time, the nature of the anti-oxidant action of ATACL is interesting: in low and medium doses (25 and 50 mg/kg), this compound prevents the development of oxidative stress, probably due to the presence of scavenger properties. In high doses (100 mg/kg), on the contrary, it intensifies free radical processes, which is an example of the classic “parabolic” nature of anti-oxidant action. Also of particular interest is the effect of ATACL on the change of the activity of the citrate synthase. Cytrasynthase is an enzymatic complex localized on the inner mitochondrial membrane that catalyzes the condensation reaction of oxaloacetate/acetyl-CoA with the formation of citrate and CoA, i.e., the first stage of the Krebs cycle (40). In addition to the metabolic role, citrate synthase is also a quantitative measure of the presence of intact mitochondria and the integrity of both outer and inner mitochondrial membranes (41). Thus, the increase of citrate synthase activity observed with the use of ATACL may result from an increase in mitochondrial biogenesis *de novo*. As a result, the registered changes promoted an increase of the ATP concentration in the hippocampus of ischemic rats. The consequence of maintaining optimal bioenergetic metabolism when the test compound is administered to animals, the restoration of cognitive functions, was noted, which is confirmed by the results obtained in the Morris water maze test. At the same time, the rats, which were treated by ATACL, spent much less time searching for a “rescue platform” than animals without pharmacological intervention. Also, the distance covered and the average speed of movement in rats receiving the test compound were higher than those in the NC group, which may indicate an increase in motivation and speed of decision-making in animals (42). As a result, the study showed that the use of ATACL at doses of 25 and 50 mg/kg (equivalent) with intraperitoneal administration, the difference from the dose of 100 mg/kg, promotes recovery of mitochondrial function and spatial memory of animals under cerebral ischemia conditions, while parenteral administration of the test compound can reduce the effective dose from 100 mg/kg (*per os*, previously obtained data) to 25 mg/kg. An advantage of test-compound over the reference may be a higher degree of effect on changes in mitochondrial function at a lower therapeutic dose (25 mg/kg versus 100 mg/kg), which will provide a better efficacy/safety ratio and therapeutic index, which is undoubtedly important for translational success.

# - reliably relative to the SO rats (*P*<0.05, v); * - # - reliably relative to the NC rats (*P*<0.05, ANOVA by post-processing with Newman-Keuls test)

**Table 1:** Effect of 4-hydroxy-3,5-di-tret-butyl cinnamic acid and the referent on changes of the pro / anti-oxidant balance in the hippocampal supernatant of rats

**Group**	**SO**	**NC**	**ATACL, 25 mg/kg**	**ATACL, 50 mg/kg**	**ATACL, 100 mg/kg**	**Mexidol, 100 mg/kg**
SOD, U/mg of protein	56,9±5,818	24,1±5,346#	33,4±6,232*	35,2±6,366*	27,3±5,251	29,5±6,957*
GPx, U/mg of protein	61,7±5,573	37,8±5,175#	47,2±5,896*	48,3±5,371*	38,3±5,356	43,2±5,646*
Catalase, U/mg of protein	24,5±1,342	11,7±2,497#	17,4±4,229*	17,2±1,12*	12,7±3,472	16,4±1,853*
H_2_O_2_, nmoles/mg	0,613±0,073	1,498±0,368#	0,958±0,041*	0,967±0,201*	1,963±0,337*	1,008±0,066*
TBARS, nmoles/mg	1,25±0,326	4,56±0,916#	3,09±0,41*	3,76±0,798*	2,73±0,781*	3,07±0,31*

Note: # - reliably relative to the SO rats (*P*<0.05, ANOVA by post-processing with Newman-Keuls test); * - # - reliably relative to the NC rats (*P*<0.05, ANOVA by post-processing with Newman-Keuls test)

SO: sham-operated animals; SOD: superoxidedismutase; NC: negative control rats; ATACL: 4-hydroxy-3,5-di-tret-butyl cinnamic acid; GPx: glutationeperoxidase; TBARS: 2-thiobarbituric acid reactive substances

# - reliably relative to the SO rats (*P*<0.05, ANOVA by post-processing with Newman-Keuls test); * - # - reliably relative to the NC rats (*P*<0.05, ANOVA by post-processing with Newman-Keuls test)

SO: sham-operated animals; NC: negative control rats; ATACL: 4-hydroxy-3,5-di-tret-butyl cinnamic acid

# - reliably relative to the SO rats (*P*<0.05, ANOVA by post-processing with Newman-Keuls test); * - # - reliably relative to the NC rats (*P*<0.05, ANOVA by post-processing with Newman-Keuls test)

SO: sham-operated animals; NC: negative control rats; ATACL: 4-hydroxy-3,5-di-tret-butyl cinnamic acid

# - reliably relative to the SO rats (*P*<0.05, ANOVA by post-processing with Newman-Keuls test); * - # - reliably relative to the NC rats (*P*<0.05, ANOVA by post-processing with Newman-Keuls test)

SO: sham-operated animals; NC: negative control rats; ATACL: 4-hydroxy-3,5-di-tret-butyl cinnamic acid

## Conclusion

Based on the obtained results, it is assumed that the use of 4-hydroxy-3,5-di-tret-butyl cinnamic acid at a dose of 25 mg/kg with parenteral administration is capable of providing the development of a neuroprotective effect realized by restoring mitochondrial function in the hippocampus of animals, which had a favorable effect on cognitive functions of ischemic animals. At the same time, the pharmacological effect from the administration of the test compound in most cases was comparable, and in some cases (the effect on the processes of cellular respiration, the activity of respiratory complexes, and citrate synthase) exceeded that from the use of the referent, ethylmethylhydroxypyridine succinate. Thus, the study showed that 4-hydroxy-3,5-di-tret-butyl cinnamic acid is a promising neuroprotective agent of complex action, the use of which is able to restore the optimal course of bioenergetic reactions and eliminate the acute PSD phenomenon, as the most serious complication of ischemic stroke.

## Authors’ Contributions

Development of the research concept, conducting the experiment, data processing, writing and editing the manuscript: PID.

## Conflicts of Interest

No conflicts of interest.
